# Perceived Factors Influencing Health-Seeking for Substance Use Among Secondary School Learners in the Western Cape, South Africa

**DOI:** 10.1177/29768357261425063

**Published:** 2026-03-30

**Authors:** Gadija Khan, Gcina Malendela, Danielle Daniels, Zaino Petersen

**Affiliations:** 1 Developmental, Capable and Ethical State (DCES), Human Sciences Research Council, South Africa; 2 Public Health Societies and Belonging (PHSB), Human Sciences Research Council, South Africa

**Keywords:** health-seeking behaviour, service accessibility, adolescent substance use, prevention and treatment gap, barriers, facilitators, self-stigma

## Abstract

**Background:**

Adolescent alcohol and other drug use represents a growing public health challenge in South Africa, particularly in the Western Cape, with early onset and high prevalence rates. Despite the urgent need for prevention and treatment programmes (aligned with SDG 3.5), a substantial treatment gap exists, with many young people failing to seek or receive adequate prevention and treatment services. Understanding perceived factors influencing help-seeking among a heterogenous group of adolescents is essential for developing youth-friendly prevention and treatment.

**Objectives:**

This research aimed to explore secondary school learners’ perspectives on factors that facilitate or hinder health-seeking behaviours in the Western Cape.

**Methods:**

A qualitative methodology was adopted, semi-structured interviews were conducted with 84 learners from 11 purposively sampled schools across 7 community clusters in Cape Town. Inductive thematic data analysis was conducted.

**Results:**

The findings highlighted perceived facilitators and barriers at individual and service levels. Facilitators and barriers reflected both the perspective of adolescents with lived experience of substance use, and general perceptions of services and adolescent help-seeking. User-level facilitators included self-determination and a desire for change, insight to seek professional help, strong preference for informal help-seeking (especially from parents), and peer encouragement and support. User-level barriers comprised self-stigma, reluctance to stop using substances, lack of knowledge of available services (among adolescents and parents), and fear of parental rejection or punishment. Service-level barriers included access constraints (geographic, financial, programme availability), unappealing and ineffective services, stigma and perceived judgement from educators and fear of confidentiality breaches within schools

**Conclusion:**

To address the treatment gap, interventions should leverage existing trust relationships (parents, peers) and develop clear pathways to professional services, be it preventative or targeted treatment. Schools must shift from punitive to supportive environments that address confidentiality and stigma concerns. Future research should examine help-seeking behaviours among adolescents with documented substance use.

## Introduction

Alcohol and other drug use among South African youth represents a growing public health challenge. A national survey of youth aged 13 to 17 years found that 12% reported consuming alcohol before age 13.^1^ Alcohol remains the most commonly used substance, with 49.6% of youth (aged 10-19 years) reporting lifetime use, 31.8% current use, and 23.0% binge drinking.^1^ Cannabis remains the most used illicit substance among youth.^1,2^ Polysubstance use is also an emerging concern, characterised by simultaneous use of multiple substances, including alcohol, tobacco, and illicit drugs.^3^ The Western Cape shows increasing adolescent substance use, as evidenced by high treatment admission rates.^4^

Adolescence is a developmental stage marked by heightened vulnerability to substance use disorders.^5^ Substance use poses significant long-term risks, given the neurological development during this developmental stage, and is associated with negative psychosocial outcomes, including poor academic performance, cognitive impairment, aggression and violence.^6^

Understanding the service-seeking landscape is important not only for adolescents who already use substances, but also for the those are at risk, seeking preventive information, or needing to support peers and family members. In line with contemporary public health framings, substance use services are conceptualised on a continuum, including primary prevention (targeting at-risk populations before use,) secondary prevention (early intervention for emerging use patterns) and tertiary prevention (treatment for established disorders).^7,8^ Adolescents seeking to access preventative information or early support may arguably face similar structural, social and phycological barriers such as stigma, confidentiality concerns, and service accessibility, as those who are seeking treatment for established use. Exploring these perceived barriers is therefore essential for developing effective prevention, early intervention, and risk-mitigation strategies. Building foundational awareness aligns with school-based prevention approaches that aim to strengthen local capacity, particularly in communities with high exposure to substance use. This study prioritises identifying perceived barriers to accessing prevention, early intervention, and treatment services among all learners in high-prevalence settings. This broader perspective is critical for informing community-wide outreach and engagement strategies before clinical intervention becomes necessary.

Although Sustainable Development Goal (SDG) 3.5 prioritises the prevention and treatment of youth substance use,^9^ a substantial service gap exists with significant underutilisation of available mental health, including substance use treatment services, among youth.^10,11^ The mismatch between service needs and service access and utilisation requires understanding factors influencing health-seeking.^10,11^ Given that adolescence shapes lifelong health behaviours, a deeper understanding of these factors would aid in the development of accessible, acceptable, youth-friendly preventative, and treatment services. Understanding perceived barriers among at-risk adolescents, before substance use escalates, can inform prevention programme design and identify modifiable barriers to early intervention. Within the treatment continuum, barriers to help-seeking occur at different stages of adolescent substance use, impacting opportunities for positive behaviour change. Identifying help-seeking barriers is therefore essential for informing successful prevention efforts for those not using, and treatment programmes for adolescents who use substances.

Literature^10-17^ suggests the service needs and utilisation gap is shaped by both user-level and service-level factors. At the user level, facilitators such as social influences, encouragement, positive attitude and religiosity have been noted. However, the barriers outweigh them, including a lack of knowledge about mental health services and their availability, perceived inaccessibility, fear of judgement, negative attitudes towards mental health, desire for self-reliance, and low perceived need for professional care.^10,11^ Adolescents’ reluctance to seek help is often rooted in psychosocial factors, such as fear of judgement and self-stigma.^12,13^

At the service level, physical inaccessibility, particularly geographic location, is a major constraint.^9,11^ Provider attitudes and behaviours severely impact health-seeking behaviour. Access is hindered when confidentiality is breached or when adolescents are treated dismissively and with hostility.^14,15^ Existing literature details these negative experiences citing moralistic language, judgement regarding their lifestyle choices, leaving them feeling reluctant to re-engage with providers.^16^ This is compounded by poor communication, resulting in feeling overwhelmed and receiving insufficient information about their health or treatment.^17^

Designing effective prevention and treatment programmes for adolescent substance use requires understanding individual, social, and contextual factors influencing adolescents’ help-seeking behaviours.^18^ Understanding the role of peer support, self-determination, and safe, confidential, and empathetic service environments is essential.^19^

This research addresses a gap by exploring facilitators and barriers to health-seeking behaviours at 2 levels: service users (learners) and service providers (care centres). It explores the lived experiences and perceptions of secondary school learners regarding substance use services within their social contexts, recognising that their views are critical for informing prevention and outreach efforts in high-risk communities. Drawing on existing literature, we anticipated key facilitators centring on peer support and non-judgemental adult mentorship while barriers include stigma and a lack of confidential, youth-friendly services. A qualitative design enables nuanced understandings of adolescent perspectives.^20,21^ This study aimed to explore the perceived factors that influence health-seeking among secondary school learners in the Western Cape. This information will guide the development of youth-centred interventions responding to the growing adolescent substance use concern in South Africa.

## Method

### Study Design, Setting, and Sampling Strategy

The study employed an interpretive qualitative framework to address the research aim. The study was conducted across 7 community clusters within Cape Town, and 11 schools were purposively sampled. The selection criteria included geographical areas characterised by high rates of crime and prevalence of alcohol and drug use in the general population. Two schools were identified within each community cluster, each served by distinct municipal infrastructure, including separate healthcare facilities, South African Police Service stations, and youth-focussed non-governmental organisations.

Conducted between 2019 and 2022, the study included learners in grades 8 to 11, grade 12 learners were excluded due to the demanding academic. Purposive sampling was employed ensured inclusion of a diverse range of learners with experiences relevant to the study objectives. Eligibility criteria required learners to be enrolled in Grades 8 to 11, aged 13 years and older. Educators assisted in identifying both boys and girls across different ages and grades to maximise variation in perspectives. To capture contrasting experiences and views, learners known/suspected to have experimented with alcohol and drugs, as well as those who were not using substances, were included. Approximately 8 learners per school were estimated sufficient to capture diverse responses. The final sample included schools and participants who agreed to participate. Information about learners who declined participation was not provided, but there was a general interest in participating in the study and obtaining parental consent for learners younger than 18 years was not difficult.

Table 1 presents the demographic distribution of the 84 participating learners, representing schools across quintiles 1 to 5. The school Quintile system in South Africa, is a ranking mechanism that serves as a proxy for the socioeconomic status of a school’s surrounding community.^22^ The ranking is based on a poverty index including income, literacy, unemployment levels, and infrastructure. Schools are grouped from Quintile 1 (poorest 20% of learners) to Quintile 5 (least poor 20%). Quintiles 1 to 3 are designated as ‘no-fee’ schools and receive the highest state subsidy, differentiating them from Quintiles 4 and 5. Most learners were aged 15 to 16 years (n = 37), with balanced gender representation. Six to eight participants per school were recruited. Thirty-three disclosed substance use; others did not or concealed use. We avoided compelling disclosure to prevent reinforcing stigma.

**Table 1.** Demographic Distribution of Participating Learners by School, Quintile, Gender, and Age Group.

School code	Quintile	Gender		Age				Sample at school	History of substance use
		Female	Male	13-14	15-16	17-18	19		
School 1	1	4	4	1	2	4	1	8	3 Explicitly mentioned
School 6	3	5	3	2	3	3		8	1 Explicitly mentioned
School 2	4	4	4		5	1	1	7	4 Explicitly mentioned
School 5	4	3	5		4	3	1	8	3 Explicitly mentioned
School 8	4	1	6		3	4		7	2 Explicitly mentioned
School 9	4	3	5	2	4	2		8	5 Explicitly mentioned
School 10	4	5	3	2	3	3		8	0 Explicitly mentioned
School 11	4	3	5	2	3	3		8	9 Explicitly mentioned
School 3	5	4	3	3	3	1		7	1 Explicitly mentioned
School 4	5	3	3		1	4	1	6	4 Explicitly mentioned
School 7	5	3	5	1	6	1		8	1 Explicitly mentioned

### Data Collection

Semi-structured interviews were conducted using an interview guide (Supplemental Material 1) developed from the research objectives, by the last author (also the principal investigator) and other research assistants; the guide was not formally pilot-tested but refined during early interviews. Interviews were conducted in English, Afrikaans, or isiXhosa with face-to-face recruitment facilitated through schools, incorporated a photo-elicitation component and were audio-recorded. Transcripts were translated into English by research assistants, with back-translation performed on 10% of interviews. Photo-elicitation offers a powerful research approach for adolescents as it fosters visual dialogue that bypasses verbal barriers, reducing the pressure of direct questioning and empowering participants to communicate experiences and perspectives in a non-threatening way.^23^ Participants were shown pre-selected images (Supplemental Material 2), which were chosen to reflect local, culturally relevant examples of commonly used alcohol and drugs present in the participants’ communities, and their perceived harmfulness. The selection was validated by the research team for cultural appropriateness and relevance. The images were presented without identifying information, enabling participants to share their own interpretations of commonly used drugs, yielding valuable insights into both personal experiences and broader community perceptions of such substances. Interviews, lasting approximately 1 hour, were conducted during school hours, in a private location on the school premises, with scheduling facilitated by principals and teachers.

The Research Ethics Committee at the HSRC approved this research (Protocol No REC 103 1/18/07/18). Permission was also obtained from the Western Cape Education Department and participating schools. All participants provided written informed consent, and minors completed an assent form in addition to parental consent.

### Analysis

An inductive thematic analysis approach^24^ was used to analyse interviews. Data were managed using Atlas.ti Software 23. Four researchers, 2 experienced researchers and 2 trainees in qualitative research methods, analysed the data; all participated in a full-day training session prior to coding to familiarise themselves with the coding framework. The analysis began with independent coding, which was collaboratively reviewed and then merged to create a comprehensive code list. Regular consensus meetings were held throughout the coding process to discuss discrepancies and refine code definitions. This iterative process involved continuously comparing new data against previously analysed content to assess the emergence of additional codes or themes and subthemes. Saturation was reached after analysing a quarter of the interviews (21), with remaining interviews confirming the thematic structure. The team critically challenged interpretations and reached consensus on the final thematic framework, ensuring a reflective analysis.

## Findings

**Figure 1.**
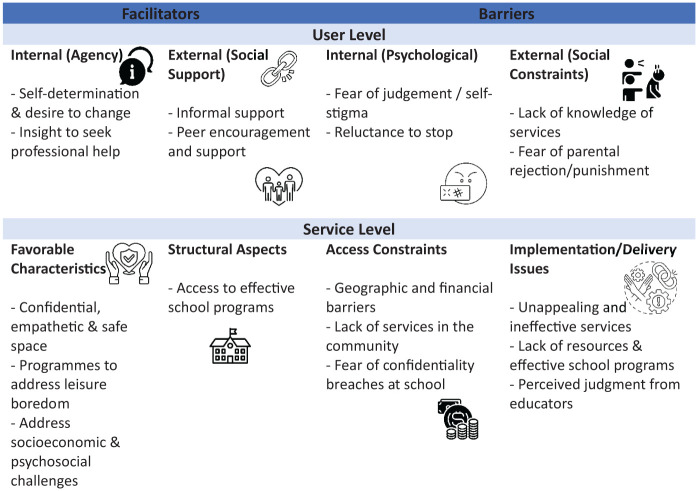
Facilitators and barriers to help-seeking for adolescent substance use behaviours. [The figure was generated through ChatGPT, after the Theme table was finalised].

The findings highlight facilitators and barriers to health-seeking behaviours at 2 levels: service users and service providers as depicted in Figure 1. The groundedness of the themes is presented in Supplemental Materials 3A and 3B. At the service user level, facilitators include preference for informal help, motivation to seek professional care, self-determination, desire for change, and peer support. In contrast, barriers included fear of judgement or self-stigma, lack of family support, limited knowledge of services, and reluctance to seek care. At the service provider level, participants identified barriers within school-based services and community programmes. Findings suggest psychosocial interventions at the service level are more effective in encouraging youth to seek healthcare when needed. Participant quotations are presented with school and learner identifiers to illustrate themes.

### User Level Facilitators

This theme encompasses 2 sub-themes: internal and external factors that facilitate adolescents’ help-seeking and/or substance use treatment. Internal factors included determination or desire to change, as well as recognition of the need for professional help. External factors involve a preference for informal help-seeking, especially from parents and peer support and encouragement.

#### Self-Determination and Desire to Change

Self-determination emerged as a key factor for seeking help and achieving treatment outcomes. Participants highlighted the role of personal conviction, rather than parental pressure, when deciding to seek treatment. They believed behavioural change is most effective when the individual is genuinely motivated.

I believe that you can change if you have the will power to change [School 2, Learner 5].

Participants noted that successful treatment requires active personal involvement in the recovery process.

But it’s up to you if you want to run away (or) If you want to stay. Until you decide now you’re alright. You don’t want to use drugs again [School 11, learner 4]

. . .if they are committed enough to doing what they want to do, they are going to counselling . . .But if you are going because you just want to go and you don’t want to actually change, then you just going to please everyone else, then it won’t help you. [School 6, Learner 2]

These quotes highlight the role of personal agency in treatment adherence, emphasising that individual commitment remains paramount from help-seeking to treatment. The responses indicate that successful behaviour change and intervention require authentic personal investment in recovery.

#### Insight to Seek Professional Help

Participants understood that substance abuse involves specialised medical and psychosocial approaches, but they could not articulate specific procedural steps when accessing care. While some learners acknowledged rehabilitation was the right setting for addressing addiction, others identified medical services such as clinics, general practitioners, or psychologists as potential entry points. Others recognised the role of law enforcement in the help-seeking process.

They need therapy. They need counselling and rehab, like if they’re really addicted to it [School 11, Learner 8]

I think they can go to a clinic and enquire if they have the correct resources and ask if the doctor can issue a letter for a rehabilitation centre, or their parents can send them to a rehabilitation centre. [School 5, Learner 1]

I will go to a social worker or police for help. [School 1, Learner 7]

#### Preference to Seek Help Informally

Participants preferred informal help-seeking, particularly from parents. Most participants cited their mothers as their primary source of support when facing difficulties related to substance abuse. This preference appears to stem from trusting parent-child relationships and learners’ perceptions of parents as their most reliable support who can connect them to appropriate services.

I would go to my mom. She would know what to do. [School 4, Learner 2]

For me I will first go to my mother or my grandmother, who I can trust with what I told them.

[School 3, Learner 4]

Because when you are at school the teachers are like our parents, for they also care about us and they’re also looking out for us. And they also want the best for us because they are laying the foundation for our future. So if you have a problem the teachers are also there [School 10, Learner 1]

#### Peer Encouragement and Support

Peer encouragement and support facilitated access to substance abuse treatment and services. Learners expressed genuine concern for peers engaged in substance use, recognising both immediate and long-term risks. They discouraged use by sharing health information, highlighting potential harm, and providing direct feedback, often motivated by empathy rather than judgement. Peer interventions aimed to rekindle the inner *‘spark’ that ‘was killed by the substance abuse’* [School 11, Learner 7].

I’ve already told them. I’ve shown them pictures of kids who use drugs. . . I showed it to them. And when I stood next to them, I got that smell. . . So I told them to stop. Because I think it’s a very bad thing [School 7, Learner 8]

### User Level Barriers

This theme consists of 2 sub-themes namely: internal (psychological) barriers and external (social constraints) barriers to accessing services.

#### Internal [psychological]

##### Self-stigma

Self-stigma emerged as a significant barrier to help-seeking among learners who use substances. Adolescents internalised negative societal perceptions of adolescents who use substances, leading to shame and diminished self-worth. This reinforced their belief that they would not receive understanding and support, discouraging them from seeking help.

Most children in the community like. . .they know they have a problem. . .they want to ask for help, it’s not always that they will ask for help because they feel like no one would help them. [School 7, Learner 2]

Participants revealed how negative or unsuccessful past experiences of approaching an educator shaped their reluctance to seek help from educators. This indicates that learners have lost trust and confidence in their educators.

I won’t actually go to my teachers at my school because I would go, but also in the back of my head you would tell me, “Don’t go” because most of them just tend to say this and that, but nothing happens. [School 11, Learner 7]

##### Reluctance to Stop

Reluctance to stop using substances was another barrier. Learners described pervasive negative influences in their communities, including peer pressure and normalisation of substance use, making quitting difficult. Although learners spend most of their time at school, participants felt that the school environment often reinforces these behaviours and lacks the capacity to prevent substance use.

Yes, because it’s like an everyday thing at my school, as most children on the school premises use it. I only feel it’s like countable even of children don’t use substances but most of the children does. [School 11, Learner 7]

Many adolescents indicated they would continue using substances, even if they faced repercussions such as school rules suspension or expulsion.

Some children even skip classes then they get suspended or expelled because they are not following the rules. And they continue smoking because the learners don’t want to stop. If they wanted to stop they would have stopped on their own but not many children are willing to complete the program. [School 2, Learner 2]

The resistance to change also emerged in interviews. If adolescents’ mindsets are not geared for change, falling back into the habit is easier

It won’t help everyone. It will only help you if you want to be helped and make a change. (But). . . as soon as we were done with these sessions they would talk about how they’re going to smoke weed when they got home. [School 4, Learner 2]

##### Lack of Knowledge of Services (Adolescents and Their Parents)

A significant barrier to accessing services was lack of knowledge about available services. Both adolescents and their parents were unaware of community-based substance abuse treatment services, with some learners surprised to learn such services existed, highlighting uncertainty about where to seek help.

I have never heard anything like that. So, what kind of help do you provide? [School 3, Learner 7]

Another participant highlighted uncertainty of where to seek help in the primary support system; when her mother was unavailable.

I would know where to go to as my mother is a very good person and she will try to help me to get help, but in the case of not having my mother, I wouldn’t know where to go because no other people would care [School 10, Learner 7]

One participant also questioned what a social worker is or what work they carry out, indicating lack of awareness of professional support for substance use addiction.

And what is a social worker? They can use drugs. I don't know ma'am, I've never heard of one. [School 8, Learner 6]

Participants reported that they have never heard of support services available, uncertainty about where to seek help, and not understanding the role of social workers.

#### External [Social Constraints]

##### Fear of Parental Rejection or Punishment

Fear of Parental Rejection or Punishment was another barrier to help-seeking. Adolescents expressed anxiety about disclosing their substance use to their parents, fearing rejection or harsh punishment. Keeping the addiction secret thus protects them from being rejected or harmed, such as physical punishment or being forced to leave the home.

Because maybe they are scared to talk about this to their mothers and fathers. Because maybe she or he would be kicked out. [School 6, Learner 4]

Parents shouldn’t beat their children when they come clean about having a substance abuse problem. Instead, they should listen to them. Because the more you beat your child, the worse he/she becomes. [School 1, Learner 4]

Another participant highlighted parental expectations that children won’t become involved with drugs. When they do use substances, it brings disapproval and disappointment, highlighting the emotional impact on adolescents.

She didn't look at me. She was disappointed. . . So nothing is worse than seeing the disappointment in her face. . ..[School 9, Learner 3]

Parents are considered a child’s primary influence and should be the first to know about their substance use. However, participants reported fear of parental rejection or punishment, which indicates adolescents’ belief that confiding in parents would invite disapproval rather than care and support.

### Service Level Facilitators and Barriers

The themes surrounding service level facilitators and barriers are crucial for understanding the challenges and opportunities in encouraging adolescents to seek help, but also the numerous barriers that impede access to the necessary services.

#### Service Level Facilitators

Service-level facilitators are factors positively influencing adolescents to seek help, particularly favourable service characteristics and structural supports. Adolescents identified features such as confidential, empathetic, and safe spaces; programmes addressing leisure boredom; and holistic interventions targeting socioeconomic and psychosocial challenges, as encouraging help-seeking. Notably, in this study, service-level facilitators was significantly less dense than the code for service-level barriers, suggesting that adolescents more readily articulated obstacles.

##### Programme to Address Leisure Boredom

Adolescents emphasised the value of services providing engaging, constructive activities as both distraction and a preventative measure against substance use. They identified leisure boredom as a key driver of substance use and suggested programmes incorporating sports and stimulating activities could facilitate engagement and recovery by offering positive outlets for their energy and attention.

I would create support groups and keep the youth busy with fun activities. [School 1, Learner 4]

I would. . .set up a programme with sports and such to keep them away from the abuse of the (substance). . .To keep them busy . . ..[School 3, Learner 1]

I will expect that they will take my mind off everything that has caused that I started using drugs [School 3, Learner 6]

##### Support from Certain Educators

For many adolescents, schools provide a consistent and accessible setting for support. Educators’ attitudes and behaviours emerged as key facilitators of help-seeking. Learners’ willingness to seek help depended on perceived trustworthiness and non-judgemental nature of specific teachers. Participants felt comfortable disclosing personal or family problems to teachers who offered empathy and practical guidance rather than criticism or public shaming. This selectivity highlights the relational and trust-based nature of adolescent help-seeking.

I would go to an educator, to the *one* I trust, because she always encourages us not to use drugs. [School 1, Learner 4]

It depends on what teacher it is. You get some teachers that you can talk to that won't judge you and who won't shame you.[School 2, Learner 2]

##### Confidential, Empathetic, and Safe Space

The foundational qualities of the service, particularly interpersonal dynamic and environment, emerged as key facilitators of help-seeking. Adolescents consistently emphasised the importance of safe, confidential, empathetic spaces. Fear of judgement was a major barrier. Participants viewed effective services as offering unconditional acceptance, patient listening, and respect for their dignity.

Firstly, they wouldn't want to be judged. They need someone that will listen to them and be patient. They need to be accepted for who they are [School 4, Learner 5]

They expect not to be judged about their addiction but to be helped. [School 2, Learner 1]

##### Holistic Interventions Addressing Socioeconomic and Psychosocial Challenges

Participants conceptualised effective services as going beyond treating substance use to addressing the underlying socioeconomic and psychosocial factors driving it. They described holistic interventions as exploring root causes, such as a lack of familial support or affection, rather than focussing solely on the behaviour itself. Adolescents also expected services to extend beyond individual treatment, offering community outreach and resource connections to help them navigate their environments and build positive futures. This desire for comprehensive support reflects their recognition of the multidimensional aetiology of substance use.

Support from family, they need to ask why the person’s using and get to the bottom of the problem [School 2, Learner 1]

They expect love, because some people don’t get love at home, and to be encouraged.[School 1, Learner 1]

I would go to the SAPS and enquire if there are any organisations that can help me within my community. . . I expect SAPS to help find outreach programmes that can help us youngers stay off the streets. [School 5, Learner 6]

#### Service Level Barriers

The findings from this study highlight several barriers that discourage adolescents from seeking help and impede access to necessary services.

##### Access Constraints

The data reveal a lack of accessible support services for learners struggling with substance use across schools, largely due to geographic and financial barriers. Participants schools should offer such support, yet current provisions fell short.

There are no programmes or services within the community or even at our school [School 1, Learner 3]

Rehab is needed but it’s not for free [School 1, Learner 5]

They need help like a facility but its far away so the ones who need help can’t get the necessary help needed. [School 5, Learner 7]

This is particularly concerning given the high prevalence of substance abuse affecting youth in these communities.

##### Lack of Services

The lack of substance use support services within communities was identified as a major barrier to accessing support. Participants reported few or no local programmes, leaving learners to rely on limited alternatives such as religious institutions, which offer moral guidance rather than practical support. Multiple respondents connect this service gap to severe, sometimes life-threatening consequences, particularly in areas with high substance use prevalence. They emphasised that many young people are not surviving their struggles due to this lack of accessible care.

There are no programmes or services within the community or even at our school.[School 1, Learner 3]

They (churches) don’t offer help; they preach about these things [School 1, Learner 4]

I don’t know of any places where I can go for help.[School 11, learner 1]

I know a few people that tend to put their time into helping young children and people, but not many support groups, as most of our people tend to look down on people who does this stuff. [School 10, Learner 7]

##### Implementation/Delivery Issues

The few existing facilities are considered unappealing, unsafe, poorly organised, and unprofessionally staffed, which discourages help-seeking. Some learners described facilities as dirty and unwelcoming, leaving them feeling unsafe. This perception undermines the trust and willingness of adolescents to engage with available services.

I don’t think it’s organized. . .even the outside, it don’t seem like a place. . .like that person would like to be at. [School 2, Learner 7]

The service is very bad. You can see it doesn’t look right at that place. There is no order. The staff is unfriendly. That is however the only place that I know of [School 2, Learner 8]

. . .it’s not a very good place. They don’t treat the people right there. It’s dirty and I don’t think anybody would ever want to go there because it’s not a place where you will feel safe or it’s a very unhygienic place also, and the employees treat the people very badly there. I don’t think they would be willing to go there either. . . [School 2, Learner 2]

Beyond concerns about physical environment and safety, participants questioned the effectiveness of existing services. Some reported relapsing after treatment, while others felt and there that schools lacked the capacity to manage substance use effectively. Disciplinary measures such as suspensions were implemented rather than supportive rehabilitative interventions.

When the school is aware that the child uses drugs, do you think they refer him for help? No. [School 10, Learner 4]

##### Perceived Judgement Experienced at School

Adolescents reported stigmatisation due to lack of trust among educators. Humiliation when trust is broken discouraged learners from reaching out to educators. This fear of stigmatisation highlights the absence of a trusted and confidential support system, exacerbating the situation and preventing learners from accessing the needed help.

Another barrier identified is the perceived judgement from educators regarding substance abuse issues. Learners feared educators would judge them and not offer substantive support, leading unwillingness to approach teachers for support.

It depends on what teacher it is. You get some teachers that you can talk to that you know won’t judge you and who won’t shame you. But then you get that teachers that you just can’t talk to, so it all depends on what kind of teacher it is. Maybe your register teachers because we grow very close to our register teachers because we are with them most of the time. So, it all depends on the teacher [School 2, Learner 2]

I don’t trust educators because they won’t be able to keep it to themselves. ([School 2, Learner 6]

I can’t trust them because if I tell them, they would tell someone else. I think that’s why some learners turn to drugs, because they are mistreated by educators. [School 5, learner 3]

Perceived judgement extends beyond information sharing, as students feared disclosing substance use would alter how teachers viewed and treated them.

Because the teacher will if I just explain what the problem is that I have a drug problem, then he will look at me differently and I don’t want that. [School 3, Learner 6]

##### Fear of Breaches in Confidentiality

Fear of confidentiality breaches discourages learners from seeking help, fearing that teachers would share sensitive information with other teachers, which could reach other learners.

Because what if the teacher tells other teachers and the teachers tell the children in the class. . . then they can mock her and stuff like that? [School 11, learner 2]

Because my friend would be scared, the teachers would tell other teachers. [School 7, Learner3]

Such breaches can lead to social humiliation and ridicule, creating major obstacles to help-seeking. Fear of information leakage and its social consequences emerged as a significant deterrent preventing students from using available school-based support services.

##### Lack of Resources and Effective School Programmes

Respondents revealed a lack of effective school resources and programmes for addressing substance use. Rather than implementing comprehensive prevention or intervention strategies, schools rely on punitive measures such as searches, testing, and suspensions, with no clear policies guiding management of substance use incidents.

They want to catch the person who uses it. If they catch the person, they give the person a letter. Then he gets suspended for 3 days or so. Then they just do it over again [School 10, Learner 4]

When they’re done smoking, and they pass by the teachers, they can smell it. The teachers don’t do anything. When they’re (learners) caught, they don’t do anything. They straighten him, or they discipline him, or something. They put him in the office. [School 8, Learner 6]

These accounts highlight ineffectiveness of current approaches, with suspensions failing to prevent recurring substance abuse. School responses are predominantly reactive and punishment-based, failing to address underlying causes or provide adequate support and referral pathways for treatment services.

## Discussion

This study provides valuable insights into the barriers and facilitators that influence the help-seeking behaviours of learners exposed to high rates of crime, violence, substance use, and who have limited access to services for adolescents who use substances. Findings reveal the complex nature of help-seeking behaviour, shaped by individual agency, and systemic socio-environmental constraints. Our findings illustrate how systemic and psychosocial barriers intersect to maintain a treatment gap, underscoring the need for integrated, youth-centred, non-stigmatising, and accessible care. Understanding these barriers across the prevention and treatment continuum that is, from primary prevention to tertiary care is necessary for developing comprehensive approaches that address adolescent needs of those who are using substances but also those who are not using, but at risk.

Intrinsic motivation and a genuine desire to change behaviour emerged as central facilitators of help-seeking. Some participants described events they experienced which resulted in a genuine desire to stop using substances, emphasising that lasting change relies on their own understanding of their situation and personal commitment to stop, rather than coercion from others. This is consistent with established behavioural change theories such as the Transtheoretical Model, where motivation and commitment to change, are based on the readiness of the individual to change that behaviour.^25,26^ This commitment is an essential first step in effective behaviour change, as found in other research^27^; however, supportive relationships, particularly with parents, peers, and trusted adults, were also found to strengthen adolescents’ confidence and self-efficacy to maintain behavioural change.^28^ This commitment and support from others are similarly important for learners who do not use drugs, to ensure that they do not fall victim to substance use.

Adolescents who reported substance use indicated a strong preference for seeking help from parents, particularly mothers, whom they viewed as reliable and non-judgemental. They also valued their role in connecting them to suitable services. Similarly, peer support emerged as another critical facilitator, with adolescents who face similar circumstances often encouraging and motivating one another to avoid substance use in an understanding manner. The importance of these informal connections to prevention and treatment should not be understated, as initial entry points to care are crucial to getting young people 1 step closer to services.^29^ International evidence has also demonstrated the effectiveness of such models, including home-based parental interventions^26^ and peer-led programmes^30^ for adolescents with mild initial substance use disorders, similar to participants in the current study.

The study also highlighted significant individual-level barriers among adolescents who used substances. Fear of judgement, internalised stigma, and anticipated punishment were major deterrents to seeking help. This fear of being labelled or discriminated against when seeking help highlights the internalised messaging linked to substance use, and how adolescents who use substances are portrayed and viewed. These negative messages prevalent in society may also deter adolescents who do not use substances from seeking information, accessing services for prevention, or supporting their peers who may contemplate using a substance. These findings mirror literature on the impact of internalised and perceived stigma in both general mental health contexts^31^ and relevance to South African settings,^32^ which must prioritise the dismantling of this internalised messaging to improve help-seeking. The focus should thus be on encouraging supportive environments aimed at positive behaviour change. Other key psychological barriers included concerns about privacy and denial of the need to cease substance use. A widespread lack of knowledge about prevention and treatment options among both adolescents and parents further constrained help-seeking, similar to what has been found in other studies.^33,34^

While findings equate effective support services as those that are confidential, empathetic, and non-judgemental, and which provide safe spaces that meet the socioeconomic contexts, service-level facilitators were mentioned less frequently than barriers. None of the learners or educators in this study referred to resources that are available to parents, learners, and educators. While parental and peer support are critical, support from educators is an important aspect of building trust in schools, as educators play an important role in creating an environment that encourages positive change. However, concerns about confidentiality breaches and moralistic judgement from educators were significant barriers for most participants with experience of using substances, as found in other school-based studies.^30^ In addition, many educators who participated in this study lacked knowledge of preventive or referral mechanisms, relying instead on punitive practices such as searches, testing, and suspension. At the community level, adolescents perceived existing services as ineffective, unappealing, and lacking basic requirements such as safety, organisation and appropriate staffing. While this is due to direct experience for some, or resulting from negative community perceptions for others, it inevitably leads to a lack of confidence in community services.

In addition to the individual and structural barriers mentioned, perceptions in this study align with studies highlighting similar contextual service delivery deficits, including individual, structural and contextual barriers, and logistical barriers, such as transport costs and long waiting times, and differences in cultural perspectives amongst service providers and young people.^32^ To be effective, prevention and treatment services must therefore be culturally tailored, non-judgemental, and youth-centred to bridge this systemic disconnect.

### Recommendations

Addressing South Africa’s adolescent substance use treatment gap requires coordinated, multi-level strategies that strengthen both informal and formal systems of care across the prevention and treatment continuum. Research is needed to explore and evaluate the integration of informal support networks into the continuum of care and pilot studies testing of parent-support groups within community health centres or schools, as described by Hlahla et al.^35^ and McDonagh et al.^36^

Further research should establish evidence and best practices for transforming youth-friendly services to align with adolescents’ expressed preferences for confidential, empathetic, and holistic care. An important area for empirical investigation includes the impact of supportive versus punitive disciplinary procedures for substance use behaviours within schools on learners’ willingness to seek help. More broadly, longitudinal studies that assess the feasibility and track outcomes of integrated intersectoral collaboration between community structures, schools, and health systems are essential.^37^

### Limitations

Certain contextual and methodological factors may have influenced the study findings. Data were collected from a sample where the substance use history of each participant was not explicitly screened or disclosed. Therefore, some participants spoke from their lived experiences of substance use, while others provided perspectives on perceived barriers and facilitators as experienced by peers or family members. The semi-structured interview guide was not pilot-tested with adolescents before data collection. However, the researchers’ familiarity with the local context and the inherent flexibility of semi-structured interviewing allowed for adaptation of questions during early interviews to ensure clarity and cultural appropriateness. Data collection took place before and during the COVID-19 pandemic and lockdown phases, which likely affected both recruitment processes and participation rates, thereby altering the broader social context within which adolescent substance use and help-seeking behaviours occur. These temporal limitations suggest that the findings reflect a particular socio-historical moment that may differ from current circumstances. Consistent with interpretive qualitative methodology, we employed collaborative consensus-building processes rather than interrater reliability testing for our qualitative coding. Readers seeking quantitative measures of coding concordance should interpret our findings with this methodological consideration in mind. Future studies should focus exclusively on adolescents with documented substance use histories to further explore how use status shapes help-seeking trajectories.

## Supplemental Material

sj-docx-1-sat-10.1177_29768357261425063 – Supplemental material for Perceived Factors Influencing Health-Seeking for Substance Use Among Secondary School Learners in the Western Cape, South Africa Supplemental material, sj-docx-1-sat-10.1177_29768357261425063 for Perceived Factors Influencing Health-Seeking for Substance Use Among Secondary School Learners in the Western Cape, South Africa by Gadija Khan, Gcina Malendela, Danielle Daniels and Zaino Petersen in Substance Use: Research and Treatment

sj-docx-2-sat-10.1177_29768357261425063 – Supplemental material for Perceived Factors Influencing Health-Seeking for Substance Use Among Secondary School Learners in the Western Cape, South Africa Supplemental material, sj-docx-2-sat-10.1177_29768357261425063 for Perceived Factors Influencing Health-Seeking for Substance Use Among Secondary School Learners in the Western Cape, South Africa by Gadija Khan, Gcina Malendela, Danielle Daniels and Zaino Petersen in Substance Use: Research and Treatment

sj-jpeg-3-sat-10.1177_29768357261425063 – Supplemental material for Perceived Factors Influencing Health-Seeking for Substance Use Among Secondary School Learners in the Western Cape, South Africa Supplemental material, sj-jpeg-3-sat-10.1177_29768357261425063 for Perceived Factors Influencing Health-Seeking for Substance Use Among Secondary School Learners in the Western Cape, South Africa by Gadija Khan, Gcina Malendela, Danielle Daniels and Zaino Petersen in Substance Use: Research and Treatment

sj-jpeg-4-sat-10.1177_29768357261425063 – Supplemental material for Perceived Factors Influencing Health-Seeking for Substance Use Among Secondary School Learners in the Western Cape, South Africa Supplemental material, sj-jpeg-4-sat-10.1177_29768357261425063 for Perceived Factors Influencing Health-Seeking for Substance Use Among Secondary School Learners in the Western Cape, South Africa by Gadija Khan, Gcina Malendela, Danielle Daniels and Zaino Petersen in Substance Use: Research and Treatment
