# Corrigendum to “Perceived Factors Influencing Health-Seeking for Substance Use Among Secondary School Learners in the Western Cape, South Africa”

**DOI:** 10.1177/29768357261474602

**Published:** 2026-09-17

Khan G, Malandela G, Daniels D, Petersen Z. Perceived Factors Influencing Health-Seeking for Substance Use Among Secondary School Learners in the Western Cape, South Africa. *Subst Use: Res Treat*. 2026;20. doi:10.1177/29768357261425063

In the above-mentioned article, the name of the author has been corrected from “Gcina Malendela” to “Gcina Malandela”.

The article has been updated to reflect the correction.

