# Supplementary material for: Perceived Factors Influencing Health-Seeking for Substance Use Among Secondary School Learners in the Western Cape, South Africa
Source: Subst Use. 2026 Mar 30;20:29768357261425063. doi: 10.1177/29768357261425063 (PMC13039619; doi:10.1177/29768357261425063)
Supplement: sj-docx-1-sat-10.1177_29768357261425063 – Supplemental material for Perceived Factors Influencing Health-Seeking for Substance Use Among Secondary School Learners in the Western Cape, South Africa [file sj-docx-1-sat-10.1177_29768357261425063.docx]

*
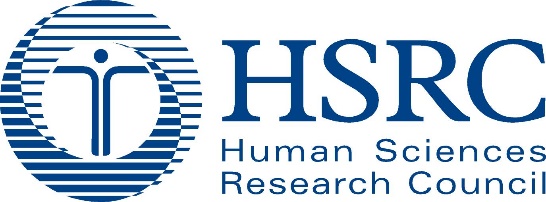

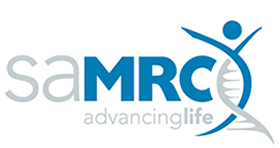
*

**ANNEXURE C: Adolescent demographic information and Interview Guide**

**Demographic information to be collected**

| Age |  |
| --- | --- |
| Gender |  |
| Current grade |  |
| School you attend |  |
| Area in which you live (If different from where your school is situated) |  |
| Who do you live with? |  |
| Recorder Number |  |

**INTERVIEW GUIDE: ADOLESCENT**

1. Do you know what substance abuse is? Could you share with me what it is? If not what do you think it is?
2. What about substance abuse do you know? Can you tell me any experiences you have had with substance abuse?
3. Do you think there is any difference between substance abuse and drugs? What are these differences?
4. Can you name places that people especially in the community use substances, where are they frequently used?
5. Can you tell me about substance use in your community, who mostly uses it (age/race/gender)?
6. Can you tell me about substance use in your school, who mostly uses it (age/race/gender)?
7. Have you ever experienced substance abuse in your family? If so can you elaborate on from who mostly?
8. Have you experienced substance use in the school? What do you think about substance use at your school and among your peers? How does this make you feel?
9. Could you tell me what you know about the following substances and what you think and how you think they make your peers feel?
10. Tobacco (cigarettes)
11. Marijuana (dagga)
12. Alcohol
13. Methamphetamine (tik)
14. Apart from the ones mentioned above do you know of any other substances (legal and illegal) if so please tell me what you know about it?
15. Do you know anyone close to you who uses any of the above or mentioned substances? how does this make you feel?
16. Apart from the ones mentioned above do you know of any other substances (legal and illegal) and please tell me what you know about it?
17. **Activity:**

**Please supply participant with substance document -** (document with all the types of substances) ask participant to Look at the pictures, the participant should go through all the alphabets:

- They should share anything they know about all the substances?
- Rate how common the use of these substances are in their community?
- How are they used?
- Who uses them (young/old/race/gender)?

1. In your view, how popular do you think is the use of the above substances by your peers/fellow learners?
2. What type of support/care/ help do you think young people who use substances need?
3. Do you know of any programmes or services aimed at supporting young people who use drugs?
4. Let’s say I was your friend, who would you go to first if I needed help with substance abuse?
5. Do you regard your parents and/or educators as key persons whom you would approach in supporting your friend regarding substance use? Please explain who and why you would go to chosen person?
6. Let’s imagine you had a drug problem or at a risk of developing a drug problem who would you turn to for assistance and why this person/structure etc?
7. What kind of help do you think young people must/can get at these centers?
8. Do you know if your parent/guardian/relative (who has a child in the school) would be interested in participating in this study? Do you mind giving me their information? **(details to be taken off recording).**
