# Supplementary material for: Perceived Factors Influencing Health-Seeking for Substance Use Among Secondary School Learners in the Western Cape, South Africa
Source: Subst Use. 2026 Mar 30;20:29768357261425063. doi: 10.1177/29768357261425063 (PMC13039619; doi:10.1177/29768357261425063)
Supplement: sj-docx-2-sat-10.1177_29768357261425063 – Supplemental material for Perceived Factors Influencing Health-Seeking for Substance Use Among Secondary School Learners in the Western Cape, South Africa [file sj-docx-2-sat-10.1177_29768357261425063.docx]

Photo elicitation – Name (including common/street name and description. If DK name, description.

| **A** | **B** |
| --- | --- |
| 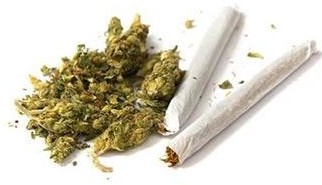 | 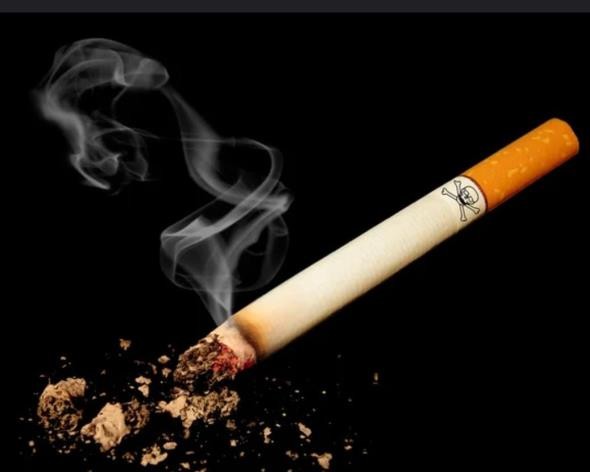 |
| **C** | **D** |
| 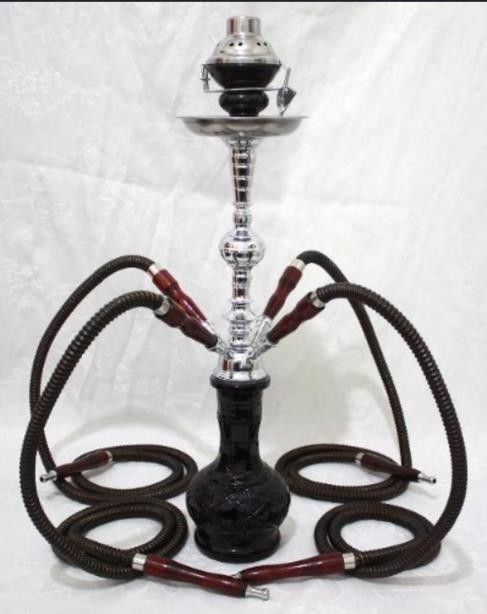 | 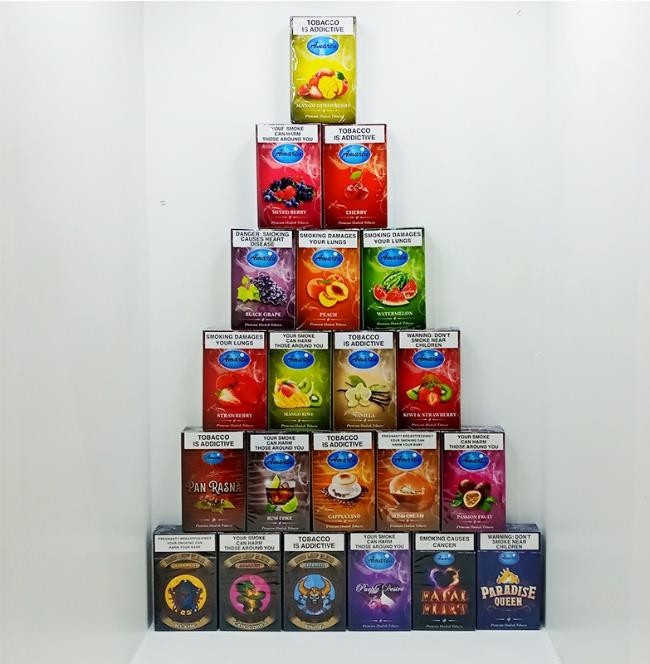 |
| **E** | **F** |
| 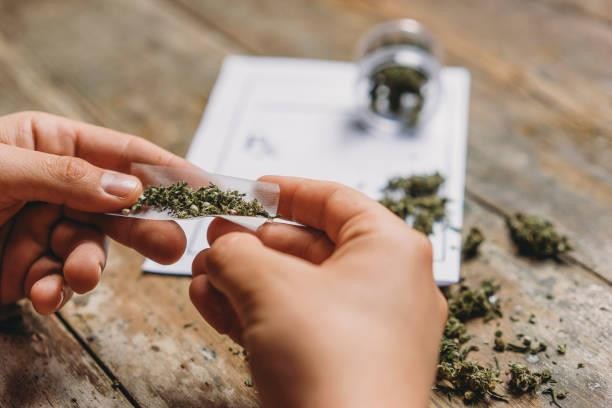 | 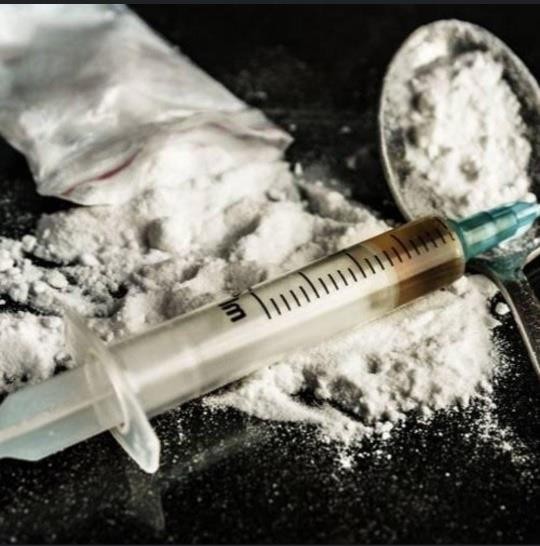 |

| **G** | **H** |
| --- | --- |
| 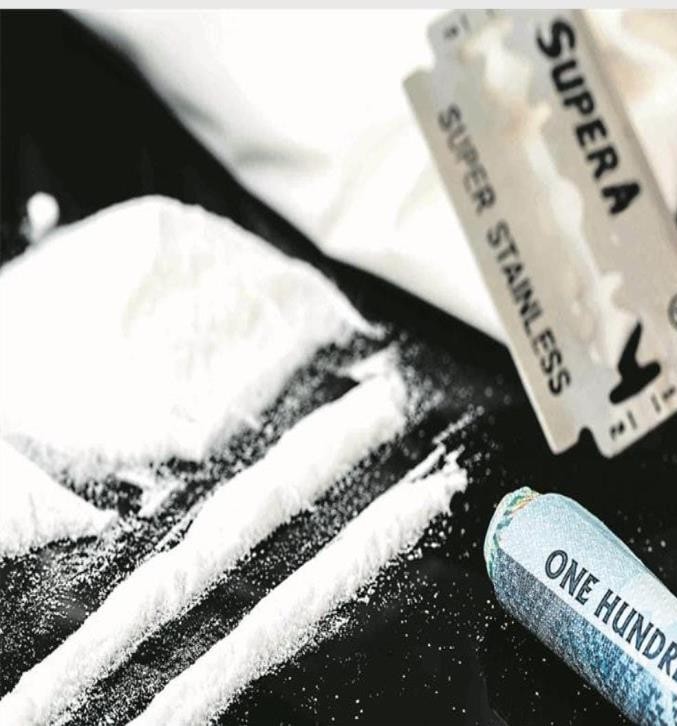 | 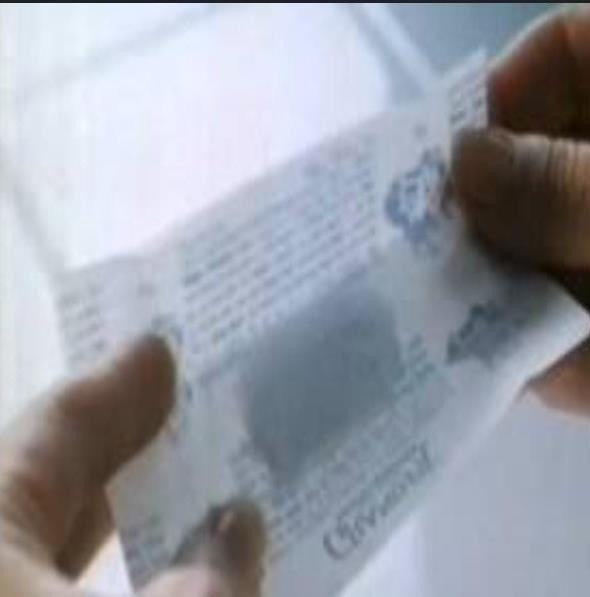 |
| **I** | **J** |
| 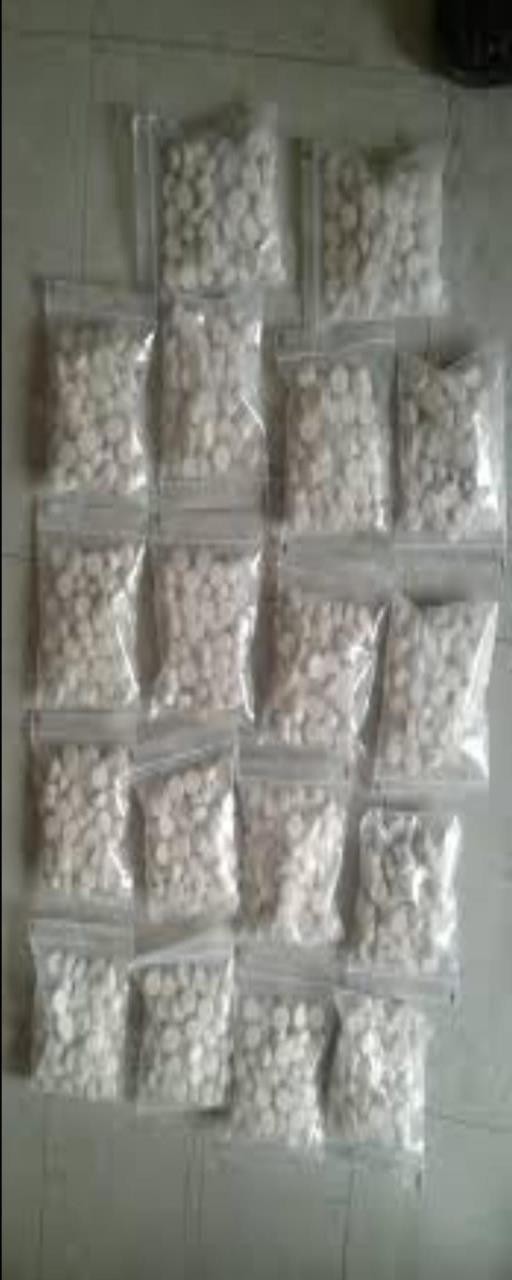 | 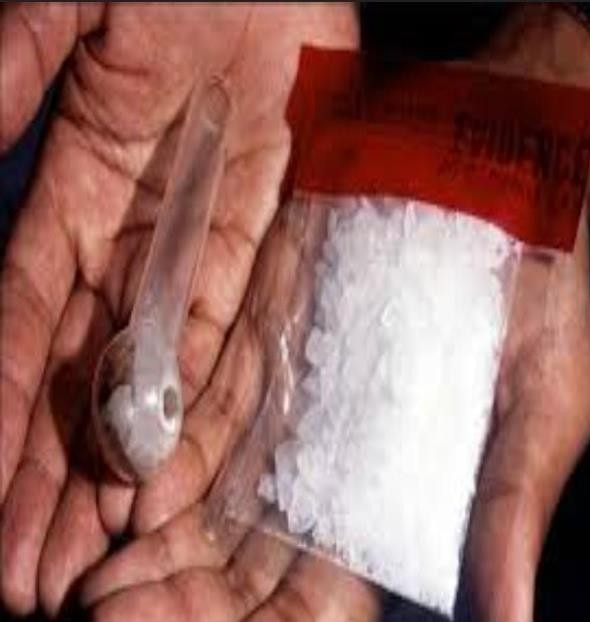 |

| **K** | **L** |
| --- | --- |
| 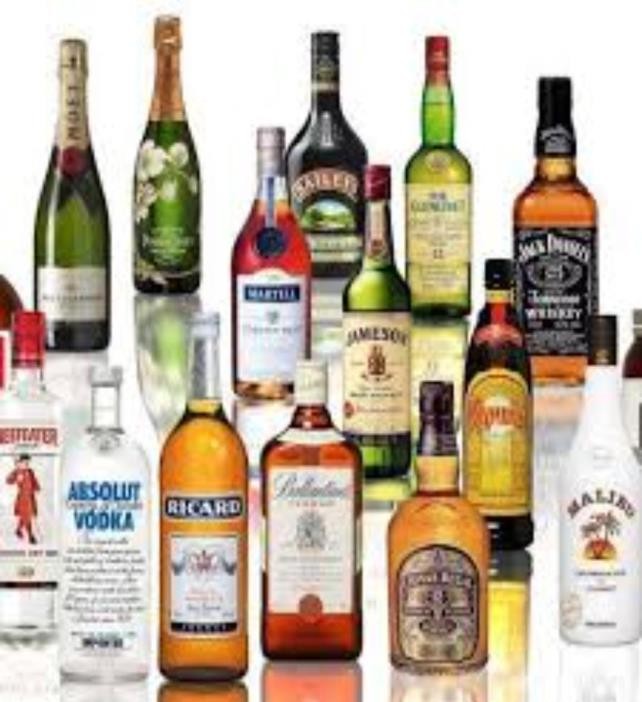 | 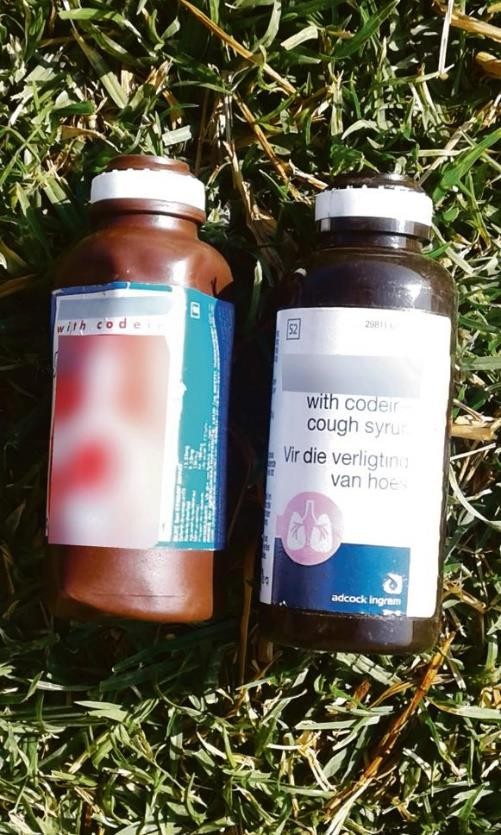 |
| **M** | **N** |
| 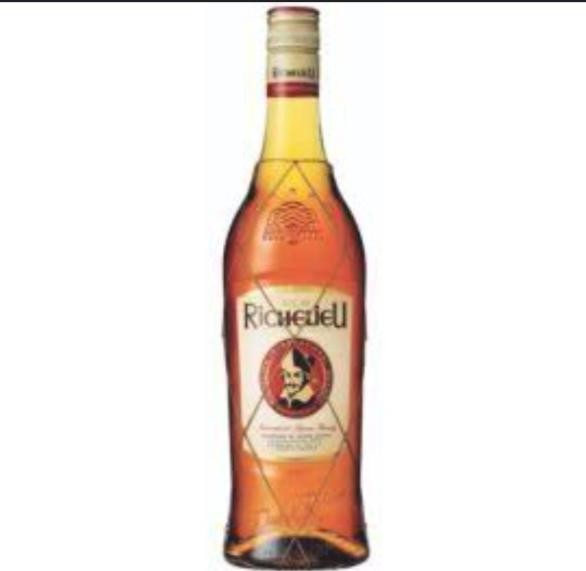 | 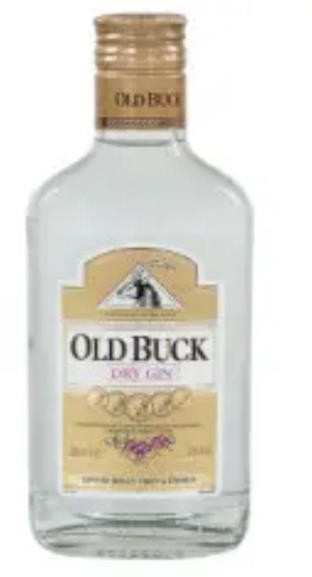 |

| **O** | **P** |
| --- | --- |
| 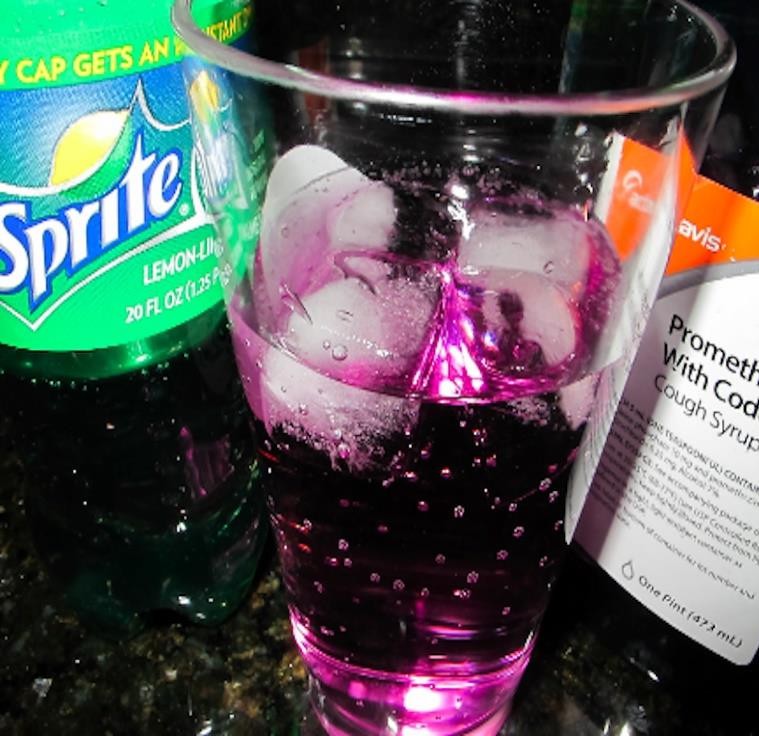 | 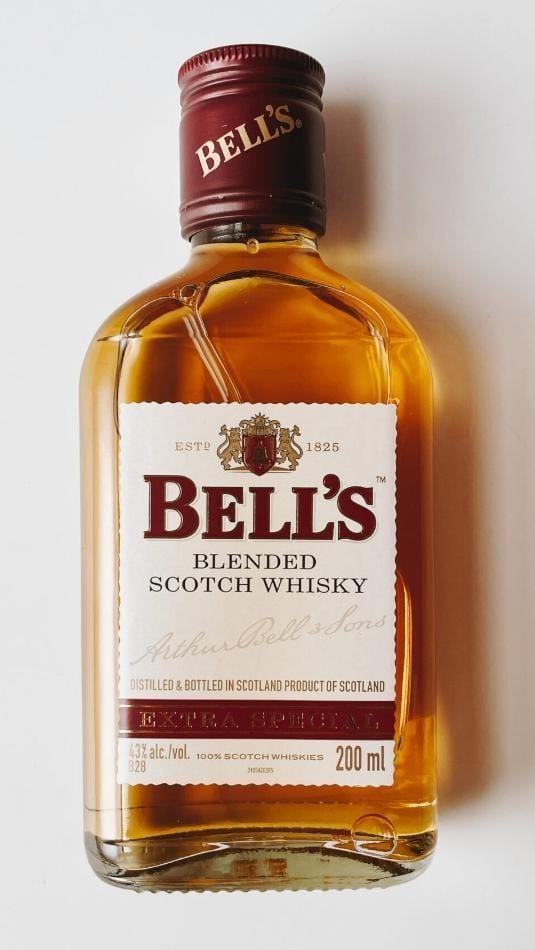 |
| **Q** | **R** |
| 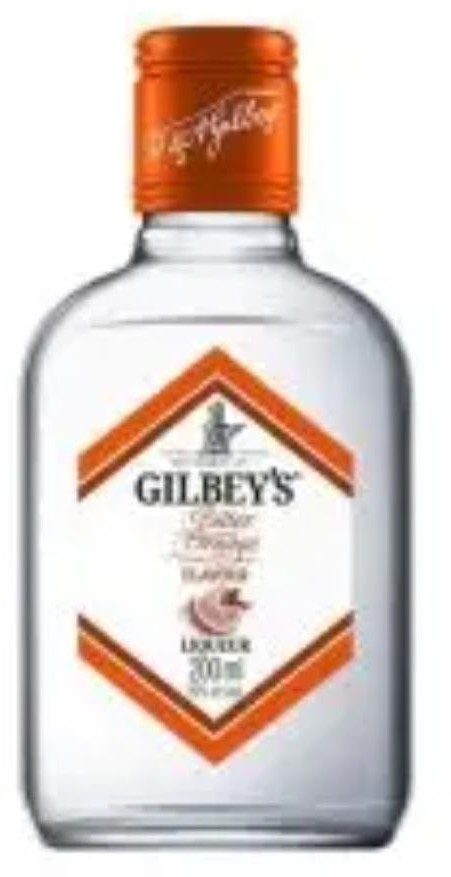 | 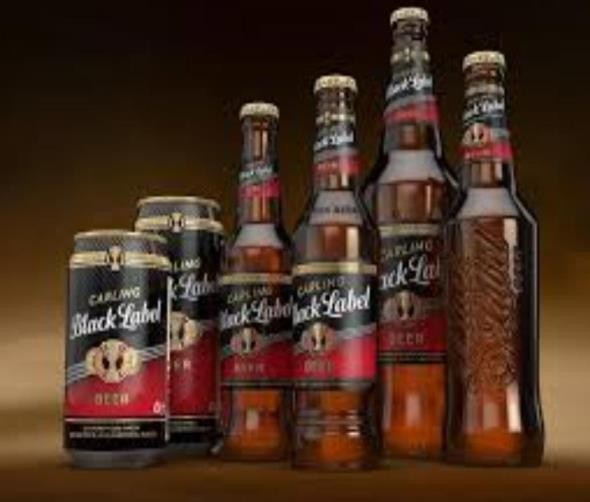 |

| **S** | **T** |
| --- | --- |
| 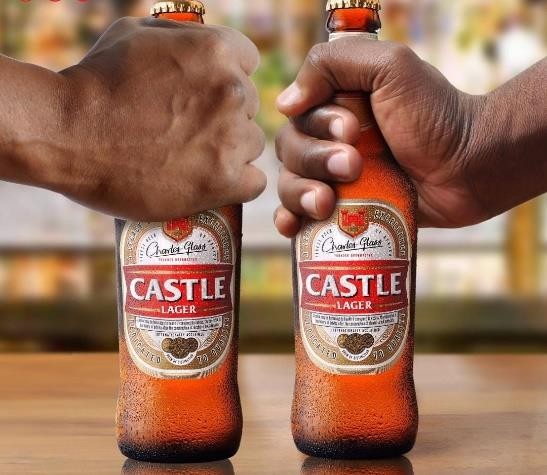 | 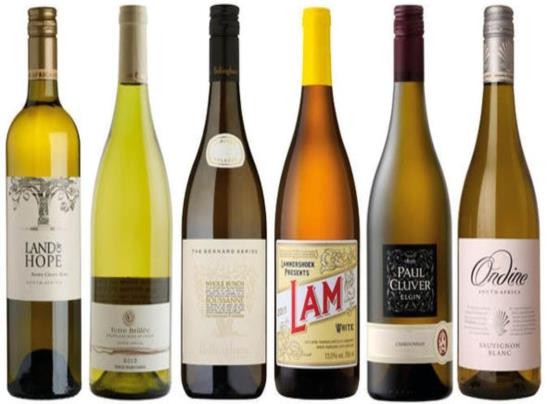 |
| **U** | **V** |
| 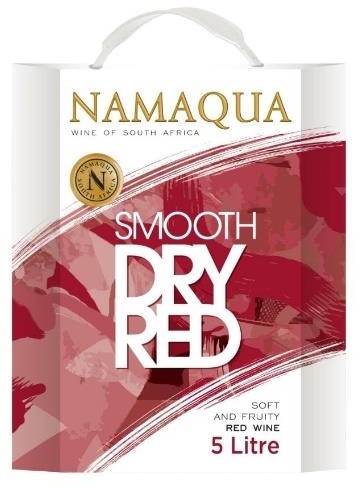 | 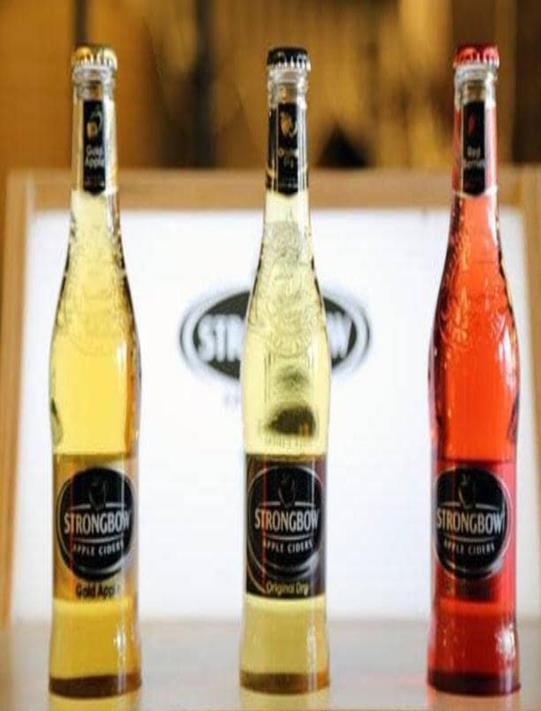 |
| **W** | **X** |
| 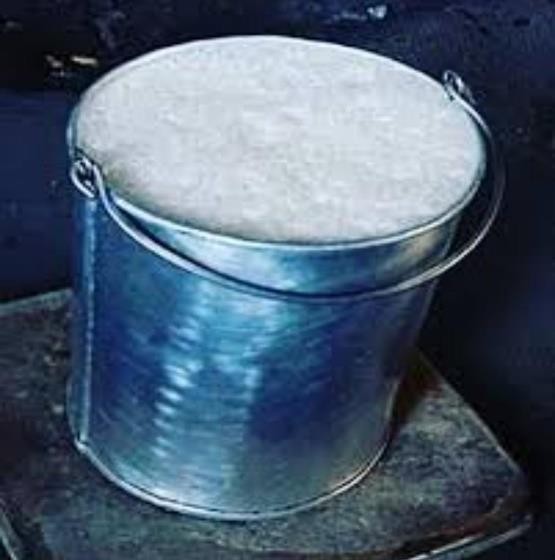 | 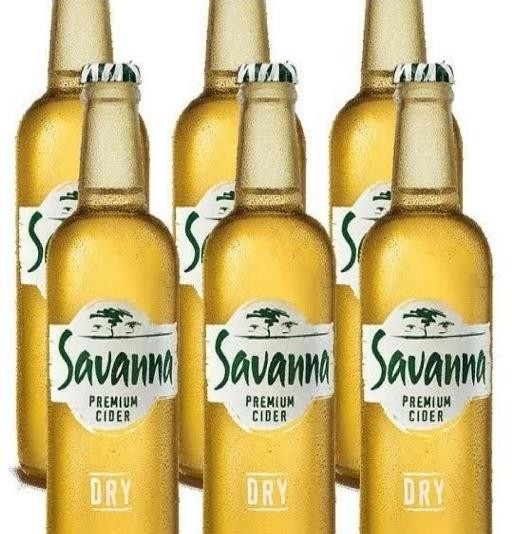 |

| **Y** |
| --- |
| 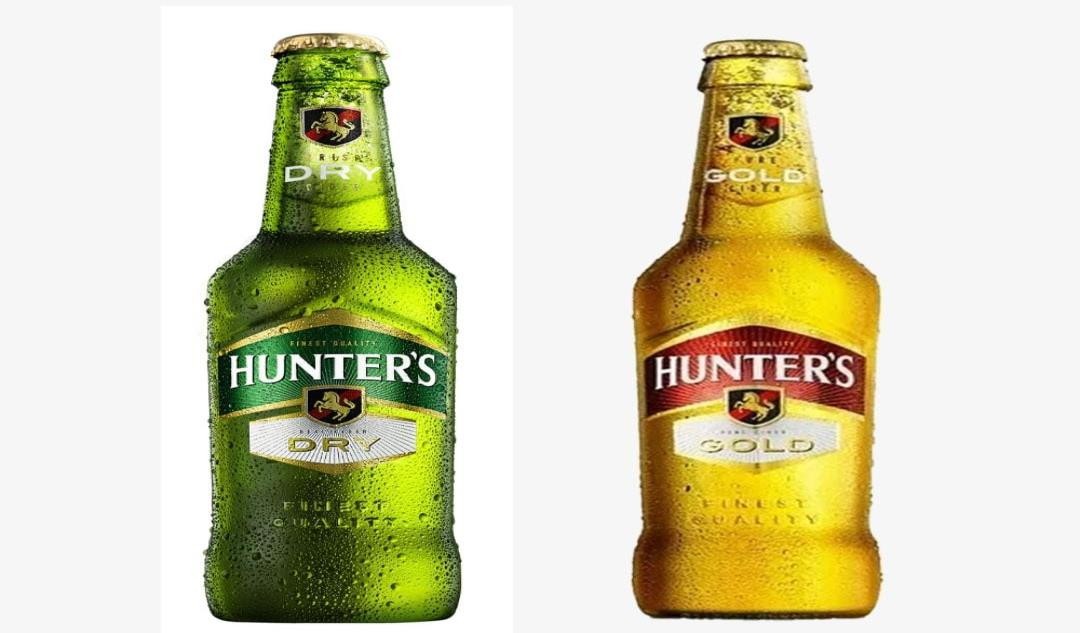 |
