# Supplementary figures and images for: Perceived Factors Influencing Health-Seeking for Substance Use Among Secondary School Learners in the Western Cape, South Africa
Source: Subst Use. 2026 Mar 30;20:29768357261425063. doi: 10.1177/29768357261425063 (PMC13039619; doi:10.1177/29768357261425063)

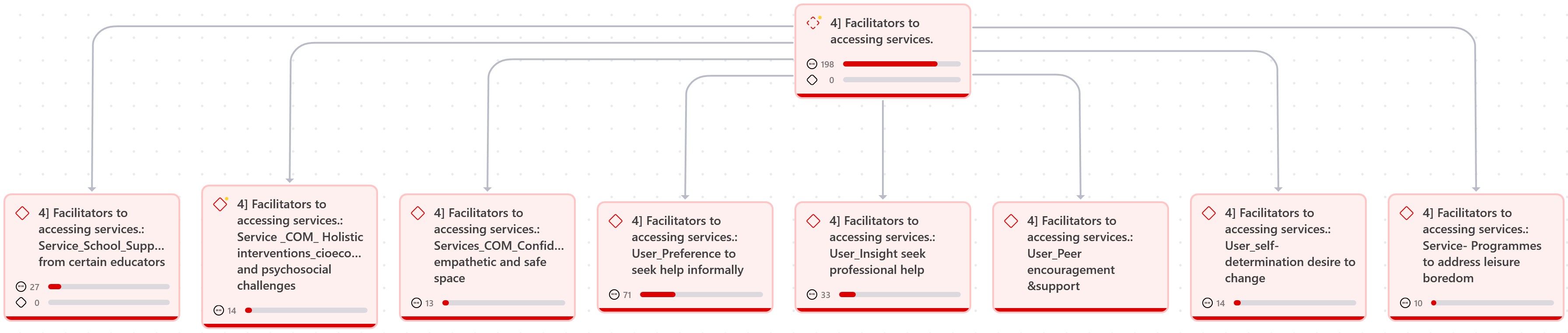

Supplement: sj-jpeg-3-sat-10.1177_29768357261425063 – Supplemental material for Perceived Factors Influencing Health-Seeking for Substance Use Among Secondary School Learners in the Western Cape, South Africa [file sj-jpeg-3-sat-10.1177_29768357261425063.jpeg]

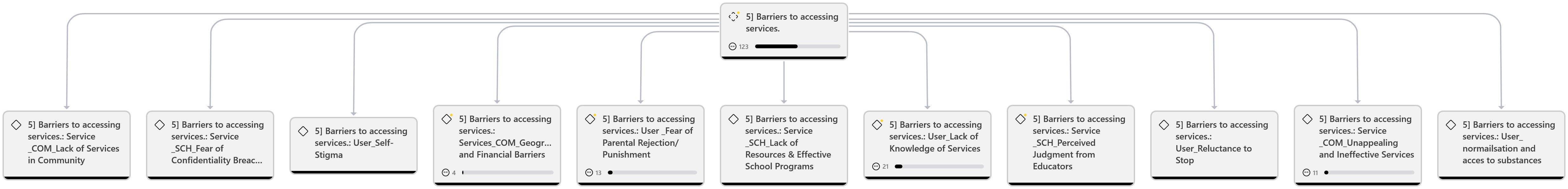

Supplement: sj-jpeg-4-sat-10.1177_29768357261425063 – Supplemental material for Perceived Factors Influencing Health-Seeking for Substance Use Among Secondary School Learners in the Western Cape, South Africa [file sj-jpeg-4-sat-10.1177_29768357261425063.jpeg]
